# Cervical Decompression and Unexpected Soft Tissue Oedema: Case Report

**DOI:** 10.5812/aapm.5048

**Published:** 2012-09-13

**Authors:** Tiscia B. Stefanutto, Stephen Gatt

**Affiliations:** 1 Gosford Department of Anaesthetist and Perioperative Care, Gosford Hospital, Gosford, Australia; 2 Prionce of Wales and Children's Hospital, Randwick, Sydney, Australia

**Keywords:** Airway Obstruction, Endarterectomy, Carotid, Spinal Fusion

## Abstract

**Abstract:**

Acute upper airway obstruction (UAO) is a life threatening complication that is well recognized after carotid endarterectomy, thyroidectomy and pharyngeal area intervention. It is not widely acknowledged that airway obstruction can occur after cervical spinal fusion surgery which was first described in 1955. There are a number of common postoperative problems which may become apparent in the short to medium term. These include: sore throat, dysphagia, hoarseness, dysphonia, recurrent laryngeal nerve palsy and soft tissue swelling.

## 1. Introduction

We report a case in which rapidly developing severe soft tissue swelling resulted in stridor and then acute upper airway obstruction shortly after an otherwise uncomplicated surgery. This required rapid reintubation and a prolonged ICU stay (nine days). We discuss the implications of the uncommon but recognized complication of massive postoperative soft tissue swelling for the perioperative management of patients after cervical spine fusion surgery.

## 2. Case Report

**Figure 1:**
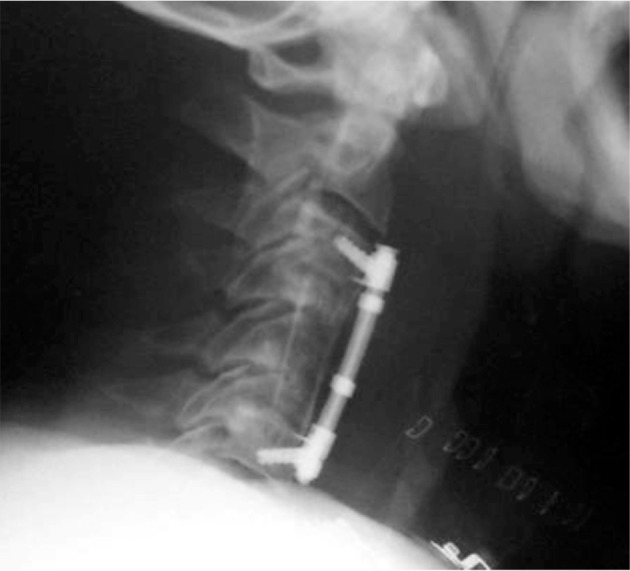
Post-Operative Lateral X-Ray Showing C3-5 Fixation With Pre- Tracheal Edema

**Figure 2:**
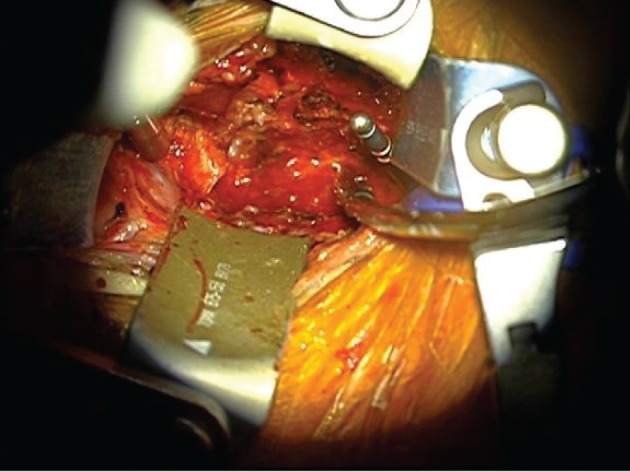
View of Retractors During Cervical Spine Surgery

The patient was a 57 year old man with cervical myelopathy of increasing severity that had developed over the course of one year. He presented with neck pain and par aesthesia in the upper left arm. There was no compromise of respiratory function. His symptoms were sufficiently severe to interfere with his work as a commercial driver. Past medical history was notable for controlled hypertension. Medication was atenolol 50 mg daily. He weighed 101kgs. Planned surgery was a C4-5 subtotal corpectomy and C3-6 bone graft fusion via a right anterior approach. He was premeditated with midazolam 2 mg iv and anaesthesia was induced with fentanyl 100 μg and propofol 150 mg and maintained with nitrous oxide in oxygen, and isoflurane 1-2 %. Vecuronium 10 mg was given after anesthesia was induced and laryngoscopy revealed a Cormack and Lehane grade I view of the larynx. The trachea was intubated with a lubricated 9.0 mm endotracheal tube and respiration was controlled to normocapnia. An intra-arterial catheter was inserted. He was positioned at fifteen degrees head up throughout surgery. Fluid replacement consisted of 2 liters of crystalloid and 500ml of Gelofusine. Surgery lasted five and a half hours and was uneventful. Estimated blood loss was 400mls. Two drains were left in situ; one superficial and one deep. The tracheal tube was removed at the end of surgery and a rigid collar was placed. He was awake and responsive with a normal ventilatory pattern. Hemoglobin saturation (by pulse oximetry) was 98-100% on 4 l/ min of oxygen via a face mask. He was transferred to the recovery suite. After one hour he complained of increasing difficulty in breathing. He rapidly became distressed and developed increasing respiratory stridor. Oxygen was administered at 100% via a reservoir breathing system. Despite this his hemoglobin oxygen saturation decreased to 90%. He became agitated. The orthopedic surgeons were summoned to exclude a hematoma or CSF collection, which they rapidly did. There was minimal drainage into the drains. Arterial blood gas analysis confirmed hypoxemia and demonstrated hypercapnia. We decided to secure his airway with an endotracheal tube. Oxygen administration was continued and optimal positioning for intubation was achieved with the collar still in place. Anesthesia was induced with thiopentone 250 mg and muscle relaxation with succinylcholine 100 mg. An anesthesiologist skilled in fibreoptic bronchoscopy was on hand and difficult airway equipment was available. At laryngoscopy we noted a Cormack and Lehane grade III view of the larynx. The epiglottis was severely edematous. There was extensive edema and swelling of the supraglottic region and the pharynx. A bougie was placed blindly into the trachea (confirmed by the tactile sensation of tracheal rings) and a 7.0 mm endotracheal tube inserted over the bougie. Positive pressure ventilation was instituted and there was no leak even with the cuff of the endotracheal tube deflated. Tube position was confirmed by x ray, and the patient was transferred to the intensive care unit. Systemic steroids and adrenaline nebulizers were started. After three days an audible leak could be heard during positive pressure ventilation and the endotrachael tube was removed. After thirty minutes he again developed marked stridor and inability to maintain hemoglobin oxygen saturation. He was reintubated and it was noted that the soft laryngeal tissues were still markedly edematous. This was confirmed by ENT who performed trans-nasal endoscopy and a cervical neck X-Ray which showed profound anterior neck soft tissue swelling (Figure 1, 2). Supportive management continued and tracheal tube was removed successfully on post-op day nine.

## 3. Discussion

Acute upper airway obstruction (UAO) is a life threatening complication that is well recognized after neck surgery such as carotid endarterectomy (1) and thyroidectomy (2). However it is not widely acknowledged that airway obstruction can occur after cervical spinal fusion surgery. Sagi et al (3) performed a retrospective chart review on 311 patients undergoing anterior cervical procedures and reported that 6.1% had an airway complication and 1.9% required reintubation. There was one fatality (3). During anterior approach spinal surgery the trachea and esophagus are retracted laterally to better visualize the anterior aspect of the cervical spine. This can lead to a number of complications: recurrent laryngeal nerve palsy, dysphonia, dysphagia, esophageal perforation, hoarseness and sore throat. Additional complications that have been reported include massive tongue swelling (4), CSF leak (5) and hematoma (6). These latter three can present as airway obstruction because of associated anatomical distortions but none were factors in the case we report here. A final possibility is allergic angioedema (7) but the temporal sequence of events makes this unlikely. Certain factors were identified in the paper by Sagi (3) et al as predisposing to acute upper airways obstruction: prolonged surgical time (> 5 h), multi-level procedures (greater than three vertebral bodies) and exposures involving C2, C3 or C4 and blood loss in excess of 300 ml. All were present in the case reported here. Epstein et al. also noted asthma, age > 65 year, obesity (> 100kgs) and severe postoperative neurological deficit (8). Prolonged and excessive deep tissue retraction can be a predisposing factor in the development of UAO. This was not an issue with our case. The combined (same day, sequentially performed) anterior and posterior procedure has a higher rate of major complications than the staged anterior or posterior procedure (9), (10). Instrumentation (plates or screws use) was shown by Sagi (3) et al not to have a significant effect on the airway complications. Our patient had two drains in situ, one deep and one superficial. These were checked by the orthopaedic surgeons who excluded haemmorhagic or CSF leak as causative. Sagi et al showed that post-operative fluid collection was not a common cause in airway obstruction. Emery et al. (11) concur. Of the patients who developed UAO only one had a hematoma and this was later in the post-operative course. Early compromise of the airway is thought to be due to the development of acute edema rather than a fluid collection. There is little consensus in the literature about the relationship between myelopathy and acute airway obstruction. However, there is consensus that there is a correlation between post-operative respiratory distress and myelopathy. Predisposing medical pathology existed in our patient He had a documented myelopathy. The time to development of respiratory distress and airway obstruction in the Emery and Sagi studies is 2 – 24 hours. Our patient became compromised at 1.5 hours. We managed this aggressively as we were in the recovery suite and decided to reintubate before the intubating conditions became too unpredictable and difficult. In light of the persistent airway edema in the post-operative period this proved to have been a lifesaving decision. Respiratory distress following cervical spine surgery is a rare complication but a potentially catastrophic one. Thus, we would like to draw attention to this potentially life threatening complication of prolonged, multilevel cervical surgery. Although UAO in these patients is a relatively rare complication it has potentially disastrous consequences and certainly a potential morbidity if not managed aggressively and definitively. Thus, the management of these patients needs to be carefully considered. Awareness of the high risk candidate based of the following factors:

1) Prolonged surgery with an operative time of > 5 hours

2) More than three vertebral bodies exposed

3) Exposures of levels C2, C3, C4

4) Combined anterior-posterior cervical spine surgery

5) > 300 ml blood loss.

When these patients are identified as high risk appropriate management should be to keep them intubated overnight as a preventative measure. A thorough airway examination with fibreoptic visualization looking for pharyngeal or tracheal swelling and lateral X-Ray to detect external neck swelling should be performed prior to attempting extubation. A leak test should be performed daily to assess improvement in a reproducible manner. Extubation should be performed over an airway exchange bougie (Cook) to facilitate reintubation and oxygenation. There must be a difficult intubation trolley available, videolaryngoscopy and surgical airway equipment (cricothyrotomy and tracheostomy) should respiratory obstruction develop. We also urge a low threshold for early reintubation should the patient appear to be developing breathing difficulties. It is imperative to educate nursing staff and medics so that they are aware of the seriousness of the development of post-operative respiratory distress in spinal surgery patients and act urgently to seek assistance to prevent disaster.
